# Psychosocial influences on pain in transgender individuals

**DOI:** 10.3389/fpain.2025.1546526

**Published:** 2025-04-24

**Authors:** Kelly S. Clemens, John Matkovic, Abby Odelson, Audrey Strain, Eric D. Wesselmann

**Affiliations:** ^1^ Department of Psychology, Illinois State University, Normal, IL, United States; ^2^ Department of Health Sciences, Illinois State University, Normal, IL, United States

**Keywords:** transgender (binary and non-binary), pain, exclusion, social pain, cognition, affect

## Abstract

Pain is an unpleasant and unavoidable part of the human experience, but the prevalence and impact of pain disproportionately impacts marginalized groups, including transgender and gender-diverse people. While there are many bases of pain, psychosocial variables, including cognitions (e.g., outcome and interpersonal expectations, social gender norms), affect (e.g., negative affectivity, emotional distress), and social factors (e.g., social exclusion) may be particularly relevant in the pain experiences of transgender individuals. The coalescence of these factors is discussed in this review, where authors specifically consider how these cognitive, affective, and social factors may contribute to pain disparities seen in transgender individuals. Patient-centered communication is presented as a potential avenue to directly mitigate the effect of these psychosocial variables on pain in transgender individuals by reducing feelings of social exclusion transgender patients may experience in the medical office, and the authors call for additional experimental research and the development of educational interventions for providers.

## 1 Introduction

Pain is a pervasive human experience; however, the prevalence and impact of pain varies significantly across different populations, with members of marginalized groups reporting greater pain occurrence and intensity than those without minority-group status (1, 2). Transgender individuals (i.e., individuals whose gender does not match their sex assigned at birth) in the United States, representing nearly .6% of the population aged 13 and older, or nearly 1.6 million individuals (3) have faced stigmatization and exclusion in the medical field and society broadly (4, 5). While research has considered binary sex and gender differences in pain for cisgender individuals [e.g., (6)], little research has considered gender differences in pain for transgender individuals or people who identify outside of the gender binary.

Pain experiences are shaped by a complex interplay of biological, psychological, social, and cultural factors (7–9). The International Association for the Study of Pain (IASP) defines pain as “an unpleasant sensory and emotional experience associated with, or resembling that associated with, actual or potential tissue damage” (10). Nociception, the process by which the body detects and responds to painful stimuli, can be altered by psychosocial factors, creating the subjective perception of pain through cognitive appraisals, learning, recalling past experiences, and active decision making (11). For example, individuals experiencing trauma or chronic stress may develop a heightened sensitivity to pain due to increased activity in pain-processing brain regions (12).

Understanding the complexity of pain experiences provides insight into how gender may shape those experiences. Gender refers to the dynamic internal sense of being a woman, man, a different gender or multiple genders, or having no gender at all, a definition endorsed by the American Medical Association (13). This differs from, but is often conflated with, biological sex, the multidimensional construct based on individuals' anatomical and physiological traits (14). Gender shapes how people navigate the world and their health due to influences of social gender norms, the socially constructed roles and behaviors that a culture deems appropriate for people based on gender (15). Gender shapes expectations, experiences, emotions, and behaviors, amongst other factors, through social gender norms. These, in turn, may shape pain experiences.

Transgender individuals then, may be uniquely impacted by the role of gender in shaping pain. However, pain research in the transgender community has been limited by systemic biases that often exclude them in medical and psychological research, and the broader marginalization of transgender individuals in healthcare. Specifically, many studies have focused on cisgender populations, failing to account for the unique physiological, psychological, and social stressors that transgender individuals face, including the effects of gender-affirming medical treatments [but see (16)]. Further, funding disparities, institutional barriers, and failure to oversample the transgender community also contribute to the underrepresentation of transgender individuals in pain research.

Beyond the influence of gender norms, transgender individuals often experience unique challenges in navigating the healthcare system (16), including fear of rejection, mistrust in providers, and providers' lack of knowledge about transgender identities (17). Furthermore, transgender individuals experience increased rates of mental health concerns (18) and overall more negative affectivity associated with minority stressors (19). These psychosocial barriers may exacerbate existing pain disparities and limit access to and effectiveness of pain treatment.

Our paper contributes to the literature by reviewing research on the interplay among cognitive, affective, and social factors on pain in other marginalized groups and extending it to transgender individuals. We also provide recommendations for future research and practical and evidence-driven ways to mitigate pain disparities for transgender individuals in the medical context.

## 2 Cognitive factors

Cognitive factors, the ways that individuals perceive, remember, think, and reason, influence an individual's experiences of pain (20), and ample evidence has emerged identifying both specific cognitive pathways [e.g., attention, self-efficacy, learning; (21)] and mechanisms by which these pathways influence nociceptive input (11). While these factors have been well-studied in cisgender individuals, we will identify those cognitive pathways which may be particularly important to the experience of transgender individuals: expectations and social gender norms.

### 2.1 Expectations

Pain responses are influenced by individuals' (1) expectations of experiencing pain [i.e., outcome expectations; (22)], (2) expectations around their social relationships and social context [i.e., interpersonal expectations; (23)], and (3) expectations regarding their ability to manage pain [i.e., self-efficacy expectations; (24)].

Interpersonal expectations, or future-focused beliefs regarding social interactions, may shape pain experiences directly or indirectly. Evidence indicates transgender individuals may avoid healthcare interactions due to expectations of discrimination (25), which may indirectly increase pain experiences due to delayed treatment initiation and disease progression. One study found that transgender individuals who delayed care due to fear of discrimination reported worse health than those who did not delay or delayed for other reasons (26). Other research found transgender patients expected to receive substandard care because of concerns about providers' ability to work with them on medical issues, and the acceptance of their identity (5).

Previous research demonstrated that beliefs about a physician's empathy and competence (i.e., interpersonal expectations) predict outcome expectations for medical treatments (27). While no known studies have considered pain-related outcome expectations specifically in transgender individuals, poor outcome expectations, stemming from the aforementioned interpersonal expectations around a transgender individual's beliefs about providers' ability to effectively treat their concerns, may negatively influence both pain experiences and the effectiveness of pain treatments. Evidence exists for differences in pain-related expectations in other marginalized groups, such as racial minorities. This may influence disparities in treatment and may also exist for transgender individuals (2).

Self-efficacy expectations have also been found to mediate gender differences in pain. Perceived physical self-efficacy and task-specific self-efficacy partly account for gender differences in pain response (28). Pain self-efficacy also affects pain behavior, pain-associated disability, and illness perceptions (29, 30). A survey of LGBT individuals in Taiwan reported lower self-efficacy and mental health help-seeking behavior among the LGBT sample compared to an age-matched general sample, and at least 28 of the 70 LGBT individuals surveyed were transgender (31). Importantly, the results were not stratified by gender identity, and therefore it is unclear whether the patterns observed among the overall LGBT sample apply equally to transgender respondents. Additional research looking at transgender individuals specifically is warranted. Relatedly, a more external locus of control is associated with greater headache disability (32) and is predictive of results of physical therapy targeting chronic pain (33). Emerging evidence suggests that transgender individuals may have a more internal locus of control than other members of the queer community (34), which may have implications for their pain experiences.

### 2.2 Social gender norms

Social norms, or the perceived, implicit rules that govern acceptable behavior, are another important pathway by which cognition may influence pain for transgender individuals. While much of the literature on gender differences in pain conflates sex and gender, evidence demonstrates that women are more pain sensitive and have a lower pain threshold than men (35). Studies have also shown that high gender identification (36) and endorsement of gendered expectations for pain (37) either wholly or partially explain this effect. Further, in a study by Robinson and colleagues (38) manipulating expectations about gendered pain tolerance eliminated gender differences. Thus, the differential influence of gender, gender norms, and sex remains in question. No known studies have examined how transgender individuals, who may have been influenced by both masculine and feminine gender norms, may experience pain because of these conflicting norms. Some research suggests that shifting salient thoughts of gender interacts with a person's tendency toward masculinity and femininity, though the nature of these interactions is complex (39). Further, other studies have identified that body image is a salient predictor of pain, and this may interact with biological sex and gender-based expectations to influence pain (40). More research is needed to understand (1) what gender norms surround pain in transgender individuals, (2) if those norms fall into existing categories of femininity and masculinity, or into novel categories, (3) what norms exist for those outside of the gender binary, and (4) how these norms influence transgender individuals.

## 3 Affective factors

The connection between negative affect and pain is well-established (41–43). Pain can be both a contributing factor to and a symptom of emotional distress. For example, while experiencing physical pain can increase a person's level of negative affective symptoms such as anxiety and depression (44), affect itself, and particularly negative affect, can also modulate an individual's pain perception, leading to increased pain (45–48). Affect presents an important dimension for understanding pain differences in transgender individuals, given their higher rates of negative affect than their cisgender counterparts (49), likely due to minority stress and increased rates in affective disorders.

### 3.1 Minority stress and pain

The minority stress model provides a framework for understanding why transgender individuals are more likely to experience negative affective consequences. Minoritized populations face external factors of discrimination more often than the majority, including social stigma, microaggressions, harassment, systemic discrimination, and violence (50). These factors significantly contribute to negative affect in marginalized groups (51–53), including transgender individuals (54, 55).

Chronic anxiety, hypervigilance, and discrimination can lead to increased levels of pain through sources such as inflammation, pain sensitivity, and migraines in transgender and other marginalized individuals (56–58). For example, racial discrimination and sexual orientation discrimination consistently predict pain (2), and sexual orientation discrimination predicts pain disparities (59). Other research suggests these pain disparities can be intensified when one has multiple marginalized identities, such as for queer persons of color (60). This is important to consider for transgender individuals, as transgender individuals are overrepresented in racial minority groups (61) and these transgender and nonbinary people of color demonstrate worse health outcomes outside of pain [e.g., (62, 63)].

### 3.2 Psychological distress and pain

Transgender individuals demonstrate elevated rates of mental health disorders (64, 65). The prevalence of depression amongst transgender and gender diverse adults was 51.3% in 2022, compared to 21.1% of cisgender individuals, and the prevalence of general mental distress in transgender individuals (38.9%) was also more than twice that of cisgender people [15.5%; (66)]. Similarly, rates of anxiety disorders (67) and posttraumatic stress disorder (68) are higher in transgender individuals (68). Evidence indicates strong connections among depression (69), anxiety disorders (56, 70), posttraumatic stress disorder (71) and pain. Thus, the disparities in mental health seen in transgender individuals may be involved in exacerbating disparities in pain.

Relatedly, transgender individuals who experience gender dysphoria, defined as clinically significant distress or impairment relating to gender incongruence (72), experience higher rates of stomach pain, panic attacks, and sensory issues (73), which may contribute to pain experiences. In comparison, those who experience gender euphoria have reduced negative affect and overall pain (56, 70), highlighting the potential influence of clinically relevant gender-related distress.

Discrimination and psychological distress are significant contributors to negative affect and chronic pain in this population. Understanding these factors is crucial for developing effective interventions that address the unique needs of transgender individuals. By considering the impact of affect on pain perception and experiencing physical symptoms, clinicians can develop targeted strategies for reducing suffering and improving quality of life among transgender individuals.

## 4 Social factors

Pain can be experienced because of physical or social injuries. Instances of social exclusion (situations in which someone feels physically or emotionally separated from others, such as being ignored) cause both hurt feelings and activates brain regions typically associated with physical pain experiences (74, 75). Further, these types of pain can have overlapping precursors and outcomes, exacerbating the individual experiences of each type (76). One common physiological explanations for this overlap involves interpersonal discrimination [a form of social exclusion; (77)] as a stressor, which thereby increases allostatic load (57), which activates the hypothalamic-pituitary-adrenal axis and sympathetic nervous system, increasing pain (78). Given the amount gender-related discrimination, microaggressions, and other forms of social exclusion transgender individuals often experience in daily life, this process may be particularly relevant (79, 80). Any exclusionary experiences within the medical context can exacerbate pain and other medical concerns.

## 5 A case for patient-centered communication (PCC)

Improving the communication between patients and physicians is an important goal among physicians. While prospective physicians receive training in PCC, research indicates emotional and empathetic responses are lacking in patient-physician interactions (81). Time spent on interpersonal communication training is often short, and studies have documented the need for improved communication (81).

Central to PCC is the concept of involving patients in treatment decision-making (82, 83). According to Naughton (82), PCC has three primary principles: allowing patients to fully explain their concerns, understanding the full context of patients' health, and working together with patients to reach shared treatment decision-making. Through these principles, PCC encourages physicians to embrace the role of “helper”, utilize open-ended questions, encourage patients to share concerns, and better understand the patient's point of view (83). When patients receive more patient-centric care, they report better symptom recovery, improved emotional health, and higher levels of treatment adherence (84, 85). They also report higher clinician satisfaction, satisfaction in overall care, and greater trust in their providers (86, 87). Thus, PCC can have an overall improvement on patient quality of life.

### 5.1 PCC and pain

Patient-centered communication has also been examined for its utility in addressing pain in patients. Research indicates that the level of PCC, and expressed empathy, can change based on whether a patient exhibits physical manifestations of pain (88). Thus, elicitation of pain and feelings from a physician is crucial to PCC. If a patient is not encouraged to fully describe pain, they may not receive the most appropriate pain care (88).

### 5.2 The case for PCC in transgender care

The current state of care for transgender patients presents opportunities for intentionally increasing PCC. For example, transgender patients often find patient-provider interactions lacking, feeling disrespected, invalidated, and judged by medical providers, or asked inappropriate questions (89–91). Many concerns expressed by transgender patients could be remedied through improved communication and empathy, with an emphasis on inviting the patient to be involved in treatment decisions (83, 91) and acting in accordance with standards of care for the health of transgender and gender diverse people (92). Positive patient-provider relationships, facilitated through PCC, may affirm transgender patients' identities, which in turn may protect against relevant feelings of exclusion and negative health-relevant cognitions (93). One strategy to better engage in PCC could involve measuring transgender and nonbinary patients' feelings of pain invalidation in order to address it in subsequent conversations (94).

Further, qualitative studies have shown transgender patients' needs are not being fully explored by physicians; when this occurs, a patient may have their pain missed, downplayed, or ignored (88, 90). Importantly, PCC may play a role in reducing patients' pain, improving their overall health, and improving the patient-provider relationship.

## 6 Conclusion

Disparities in pain experiences and management among transgender individuals represent a critical and underexplored area of healthcare inequity. Previous research has highlighted the psychophysiological underpinnings that explain the connections among cognitions (11), emotions (95), social exclusion (78), and pain. In this article, we highlight how these factors converge for transgender individuals, potentially explaining disparities in pain experiences.

Transgender individuals often report negative healthcare experiences due to discrimination and stigma (96), contributing to negative expectations about future healthcare encounters, exacerbating pain and delaying treatment. The influence of social gender norms around pain, often rooted in binary conceptions of masculinity and femininity, complicate the understanding of pain experiences as transgender individuals may experience conflicting norms. Minority stress and psychological distress, which are common in transgender individuals, can also worsen pain experiences. Finally, social exclusionary practices and microaggressions not only increase pain but also erode trust in healthcare providers, further marginalizing transgender patients.

The overlap between physical and social pain underscores the importance of addressing interpersonal discrimination and fostering inclusive environments within medical contexts. We propose that patient-centered communication (PCC) is one pathway to addressing these factors; recommending physicians invite patients into the decision-making process, make patients feel welcomed through inclusive and gender affirming language, avoid making gendered assumptions about medical care, and make efforts to understand the full context of the patient's health thereby reducing negative cognitions, affect, and experiences of social exclusion. These actions are recommended to improve patient-provider relations, build trust, and reduce feelings of patient-discrimination.

While a *basis* for understanding the mechanisms of the differential pain experiences in transgender and other gender-diverse people exists, there is no extant experimental research that examines these mechanisms and contextual differences. This gap needs to be addressed by future research. Additionally, care should be taken when conducting research to ensure consistent, precise language is used so research may be generalized to the broader transgender community, and ultimately, help improve transgender individuals' pain experiences. Uniformly applying terms such as gender identity, gender presentation, and adherence to gender role norms is crucial to improving access to this topic throughout the literature. Addressing these cognitive, affective, and social factors requires systemic intervention to reduce stigma. Providers need evidence-based educational interventions to ensure that all those involved in patient care are knowledgeable about the issues that transgender patients face and so that they are equipped to deal with these issues in a patient-centered way.

Medical providers and researchers should intentionally represent the experiences of transgender individuals, particularly transgender people of color. Recruiting these samples presents a challenge due to their small proportion within the general population and the historical distrust that both transgender individuals and people of color may have toward researchers. Nevertheless, we emphasize the importance of making concerted efforts to include these populations in research. We also encourage researchers to capture both gender and sex variables and intentionally oversample and recruit this population when exploring the influence of psychosocial factors on pain using previously validated measures. Future research is needed to determine and validate modifications to existing tools in transgender populations or develop new cognitive pain assessments aimed at those outside the gender binary to provide more accurate pain representations experiences in transgender populations.

Addressing pain disparities in transgender individuals demands a multifaceted approach that integrates cognitive, affective, and social perspectives with empathetic and inclusive healthcare practices, ensuring that transgender individuals receive equitable, affirming, and effective pain care. By doing so, the medical community can take a meaningful step toward mitigating health inequities and improving quality of life for transgender patients.
